# Propagation-based phase-contrast synchrotron imaging of aortic dissection in mice: from individual elastic lamella to 3D analysis

**DOI:** 10.1038/s41598-018-20673-x

**Published:** 2018-02-02

**Authors:** Gerlinde Logghe, Bram Trachet, Lydia Aslanidou, Pablo Villaneuva-Perez, Julie De Backer, Nikolaos Stergiopulos, Marco Stampanoni, Hiroki Aoki, Patrick Segers

**Affiliations:** 1 IbiTech – bioMMeda, Ghent University, Ghent, Belgium; 2 STI IBI-STI LHTC, Ecole Polytechnique Federale de Lausanne, Lausanne, Switzerland; 3 Paul Scherrer Institute, Villigen, Switzerland; 4 Department of Cardiology and Center for Medical Genetics, Ghent University Hospital, Ghent, Belgium; 5 Cardiovascular Research Institute, Kurume University, Kurume, Japan; 6 Present Address: CFEL (DESY), Villigen, Germany

## Abstract

In order to show the advantage and potential of propagation-based phase-contrast synchrotron imaging in vascular pathology research, we analyzed aortic medial ruptures in BAPN/AngII-infused mice, a mouse model for aortic dissection. Ascending and thoraco-abdominal samples from n = 3 control animals and n = 10 BAPN/AngII-infused mice (after 3, 7 and 14 days of infusion, total of 24 samples) were scanned. A steep increase in the number of ruptures was already noted after 3 days of BAPN/AngII-infusion. The largest ruptures were found at the latest time points. 133 ruptures affected only the first lamella while 135 ruptures affected multiple layers. Medial ruptures through all lamellar layers, leading to false channel formation and intramural hematoma, occurred only in the thoraco-abdominal aorta and interlamellar hematoma formation in the ascending aorta could be directly related to ruptures of the innermost lamellae. The advantages of this technique are (i) ultra-high resolution that allows to visualize the individual elastic lamellae in the aorta; (ii) quantitative and qualitative analysis of medial ruptures; (iii) 3D analysis of the complete aorta; (iv) high contrast for qualitative information extraction, reducing the need for histology coupes; (v) earlier detection of (micro-) ruptures.

## Introduction

Aortic dissection (AD) is defined as a disruption in the media leading to a separation of the layers of the aorta, called elastic lamellae^1^. The separated media is accompanied by at least one a tear in the intimal layer of the vessel. As blood is entering the media, a separate and parallel (false) channel is formed^1^ making AD a life-threatening disease with high mortality^1^. From a biomechanical point of view, aortic dissection is assumed to result from an imbalance between the mechanical stresses (invoked by the pulsatile blood flow and the mechanical anchoring and tethering of the aorta on organs and surrounding tissues) and the tissue strength^2^. At this moment, we have an incomplete understanding of the (mechano-) biological processes leading up to this aortic wall failure. It is commonly accepted that dissections originate from a tear in the inner layers of the aorta followed by intramural layer separation. However, such an intimal tear is not always present on medical images and it is unknown to what extent side branches or vaso vasorum play a role in disease initiation^3^. In order to improve the diagnosis and early detection of AD and to help with risk assessment and stratification, the mechanisms of dissection initiation and early arterial remodeling need to be fully understood. This is very difficult to study as patient data on the early events is often lacking and incomplete, especially on the micro-structural level of individual lamellae. The use of pharmaceutically-induced mouse models of aortic dissection allows for a controlled and fast induction of intimal and medial tears^4^. In a recently published paper we documented an *in vivo* dissection, which showed that these events take place within a time span of several hours in these mice^5^.

Most imaging techniques for small animal research, however, do not provide the image quality and resolution that is required to analyze aortic wall pathology with the necessary detail^6^. Nevertheless, the differentiation between healthy tissue and diseased tissue is pivotal in understanding the initiation and progression of cardiovascular disease - both clinically and pre-clinically. That is why, ever since the landscape of medical imaging and diagnostics was changed dramatically with the discovery of X-rays by Roentgen in 1895^7^, there has been a constant search to improve resolution and image contrast^8^.

Mice have a thoracic aortic diameter of 0.8–1.1 mm, a wall thickness of 50–100 µm and individual elastic lamella that are around 3 µm thick^9–11^. Using *in vivo* µ-CT, an isotropic pixel size up to 50 µm can be achieved^12^. However, this technique does not allow for a visualisation of the aortic wall. So information about the separation of the medial layers - which is imperative for AD research - is lacking. Such information could be obtained with histological coupes (slice thickness of 4 µm), but histology is very labour-intensive, which practically impedes a 3D analysis^13^. Even more, tears, fractures, compressions and folds are frequently induced in the process of creating histological coupes^14^. *In vivo* µ-MRI provides excellent soft tissue contrast, but its axial resolution is insufficient for detailed analyses (of about 100 µm in-plane)^15^. Despite being able to offer 3D images, high-frequency ultrasound (in-plane pixel size ~ 15 µm and through-plane pixel size ~ 35 µm) is a technique that is highly operator dependant^16^.

The last couple of years, we have experimented with synchrotron-based techniques to explore how improved imaging methods could provide deeper insights in preclinical models of cardiovascular disease. Using differential phase contrast X-ray tomographic microscopy (PCXTM) and grating interferometry, a pixel size of 6.5 μm has been achieved^16–19^. Recently, multiphoton excitation fluorescence microscopy in combination with computational image processing has been used for the visualization of individual elastic lamellae^11^. Although this technique provides a 0.5 µm resolution, the aortic sample needs to be cut longitudinally, which limits 3D analysis. Furthermore, there is light absorption and scattering throughout the different layers of the aorta, resulting in loss of signal at about 60 µm, impeding complete aortic wall visualization^11^. Others made use of X-ray microscope techniques to visualize medial and adventitial layers in *ex vivo* rat common carotid artery (CCA) with a sub-micron resolution (voxel size of 0.5 µm)^20^. With this technique, they were able to show the individual elastic layers in the CCA^20^.

One of the key discoveries based on our earliest PCXTM studies was that ApoE^−^/^−^ Angiotensin II (AngII)-infused mice develop distinctly different lesions in the ascending and the thoraco-abdominal aorta. In the thoraco-abdominal aorta, all lesions included one or several medial tears, often located on the left and ventral side of the ostium of the celiac artery^16^. These tears, assumed to be the initiating events, resulted in important lesion variability: in some mice the medial tear led to an adventitial dissection, which in turn caused small side branches to rupture and led to the formation of an intramural hematoma. In other mice, the medial tear is stable and no hematoma is formed^19,21^. Since this pathology resembled the AD progression observed in human patients these lesions have been termed ‘dissecting aneurysms‘. Taking advantage of micro-leaks of contrast agent that had been injected *in vivo* prior to sacrifice, PCXTM images also allowed us to visualize micro-ruptures in the media after only 3 days of AngII infusion. Unfortunately single elastic lamellae were not resolved on contrast-enhanced PCXTM images, so only the largest micro-ruptures – termed medial tears - could be detected. In the case of ascending aortic aneurysms, AngII infusion led to far less variability in lesion morphology than what had been observed in the abdominal case. Unlike thoraco-abdominal tears, medial tears in the ascending aorta did not affect the entire media but only the luminal layers^19^. The largest tears were situated on the outer convex aspect of the aorta and interlamellar hematoma was observed to accumulate at the outer, adventitial side of the media after 3 days of AngII infusion. However, since single lamellar ruptures could not be visualized the source of these micro-hematomas could not be established unequivocally.

In the recent past the use of ApoE^−^/^−^ deficient mice for pre-clinical aneurysm research has been questioned, partly due to the synchrotron based findings mentioned above^5^. Several alternatives have been proposed, such as the administration of AngII with the intraperitoneal injection of anti-TGF beta antibodies^16^ or the combination with β-aminopropionitrile monofumarate (BAPN)^22^. While our first synchrotron-based research showed little to no difference between anti-TGF-beta triggered lesions and traditional AngII-infused lesions^16^, BAPN mice have never been investigated in similar detail. BAPN is an inhibitor of lysyl-oxidase that plays an important role during elastogenesis and synthesis of collagen as it mediates cross-linking of tropo-elastin monomers to form an insoluble functional elastin polymer and cross-linking of tropocollagen to form a collagen fibril^23^. These collagen and elastin cross-links contribute the most to aortic compliance and resilience, so the disruption of lysyl-oxidase might lead to mechanical instability and fragility of the aortic wall and, consequently, to cystic medial degenerations^22,24^. In combination with AngII, a blood pressure elevator, AD and aneurysms are induced in these mice^22^. Research has indicated that, compared to other mouse models, the BAPN + AngII mice are more prone to develop medial ruptures^25–27^. This observation and the fact that the BAPN + AngII infusion leads to aneurysm- and dissection-like symptoms ^22,25,26^may explain the recent surge in research papers that use the BAPN mice and rats as a model for aneurysms^25,27^, dissections^22^, or both^23^.

In this work we propose to advance the state of the art in phenotyping the aorta in mouse models for aortic disease through a combination of two approaches. First, we use propagation-based phase-contrast imaging rather than grating interferometry that was used in prior synchrotron experiments, on mouse aortic tissue. We recently published a paper regarding the discussed imaging techniques (under review at the moment of publication)^28^. Combining the advantage of a full 3D analysis with a small effective pixel size (1.625 μm), propagation-based phase-contrast imaging allows for the 3D visualisation of individual lamellae that prior techniques lacked. Second, we apply this novel technique to investigate the initiation and development of ascending and thoraco-abdominal dissection disease in n = 10 BAPN/AngII-infused mice. The focus of the study was to (i) explore the possibilities of single lamella visualization offered by propagation-based phase-contrast imaging, and (ii) provide a qualitative and (to a lesser extent) quantitative description of BAPN-induced micro-ruptures and medial tears in the ascending and thoraco-abdominal aorta.

## Results

### Lesion incidence and size

**Figure 1:**
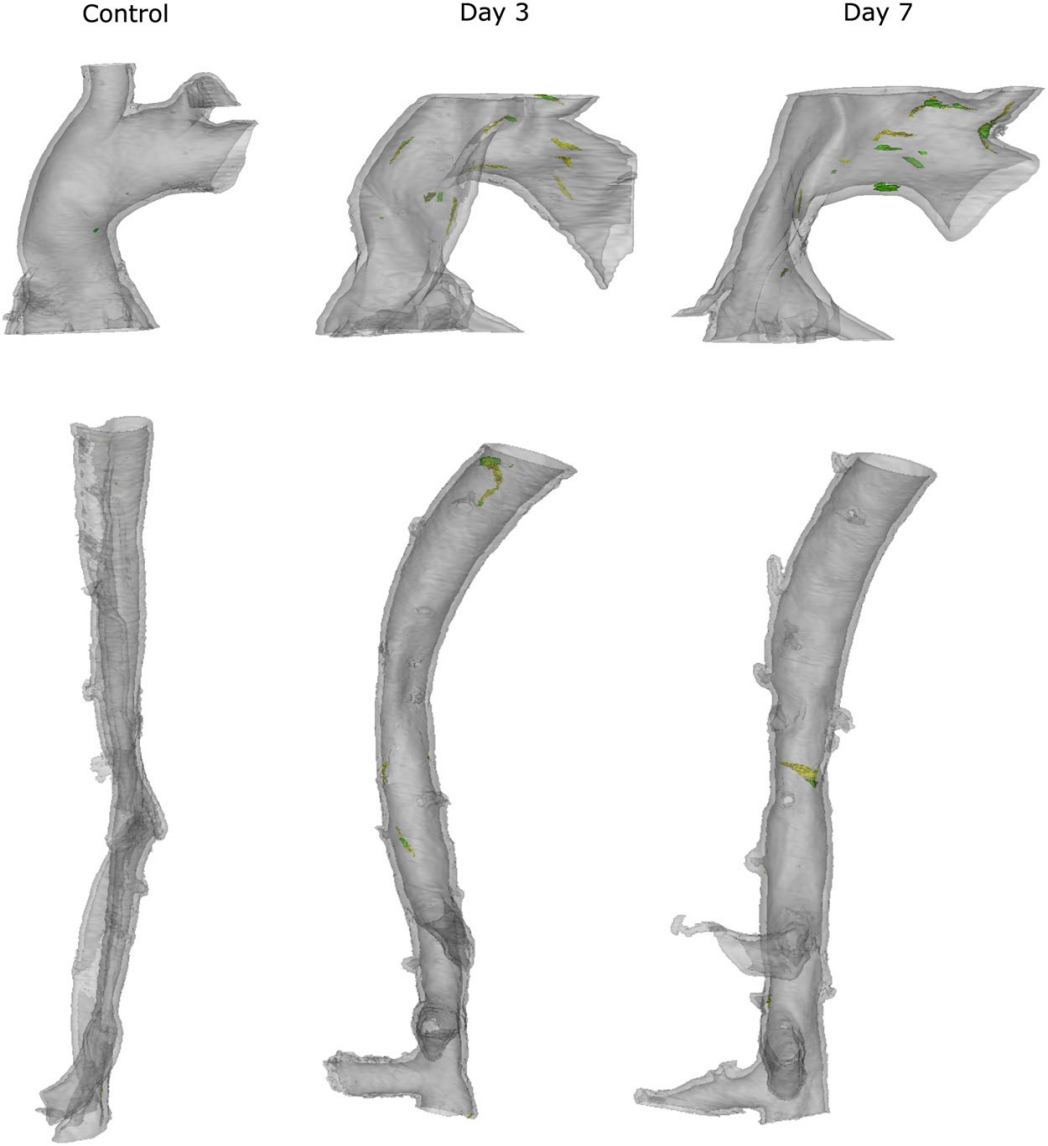
3D presentation representation of ascending (top) and thoraco-abdominal (bottom) aorta of control animal (J3), day 3 (ascending sample = J5, thoraco-abdominal sample = J4) and day 7 (J7). Grey transparent = media, yellow = L1 rupture, green = ML rupture.

**Figure 2:**
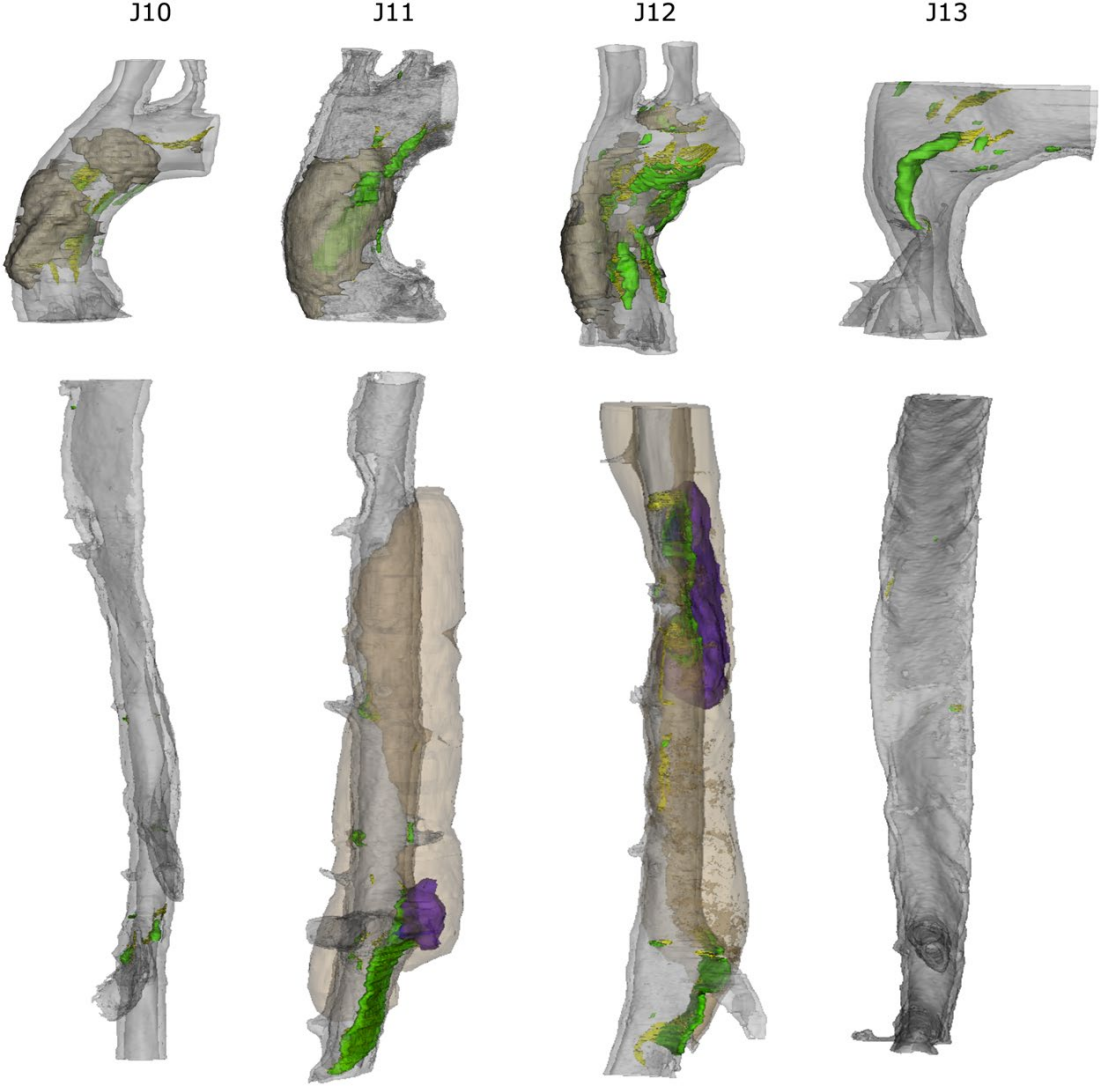
3D presentation of ascending (top) and thoraco-abdominal (bottom) aorta of day 14 animals. Grey transparent = media, yellow = L1 rupture, green = ML rupture, brown transparent = hematoma, purple = false lumen.

None of the mice died of complications before sacrifice. Representative examples of the 3D images of the different samples can be found in Fig. 1 (control animals and early time points) and Fig. 2 (day 14 animals). For the ascending aorta samples, we found a total of 63 ruptures in the first medial layer and 90 interruptions that affected multiple layers. In the thoraco-abdominal samples, a total of 70 L1 and 45 ML ruptures were found. The incidence of medial ruptures changed over the different treatment groups, as can be seen in Table 1.

**Table 1:** Overview of rupture incidence between treatment groups (mean ± SD).

Treatment group	ASCENDING AORTA			THORACO-ABDOMINAL AORTA		
	n	# L1 ruptures/n	# ML ruptures/n	n	# L1 ruptures/n	# ML ruptures/n
Control	3	0.7 ± 0.94	2.0 ± 1.63	2	2.0 ± 2.00	1.0 ± 1.63
Day 3	3	6.0 ± 2.16	5.3 ± 2.36	3	4.7 ± 0.94	2.7 ± 2.36
Day 7	2	6.5 ± 0.50	8.5 ± 0.50	3	8.3 ± 3.09	3.7 ± 0.50
Day 14	4	7.5 ± 1.12	12.8 ± 4.92	4	6.8 ± 2.49	6.0 ± 4.92

**Figure 3:**
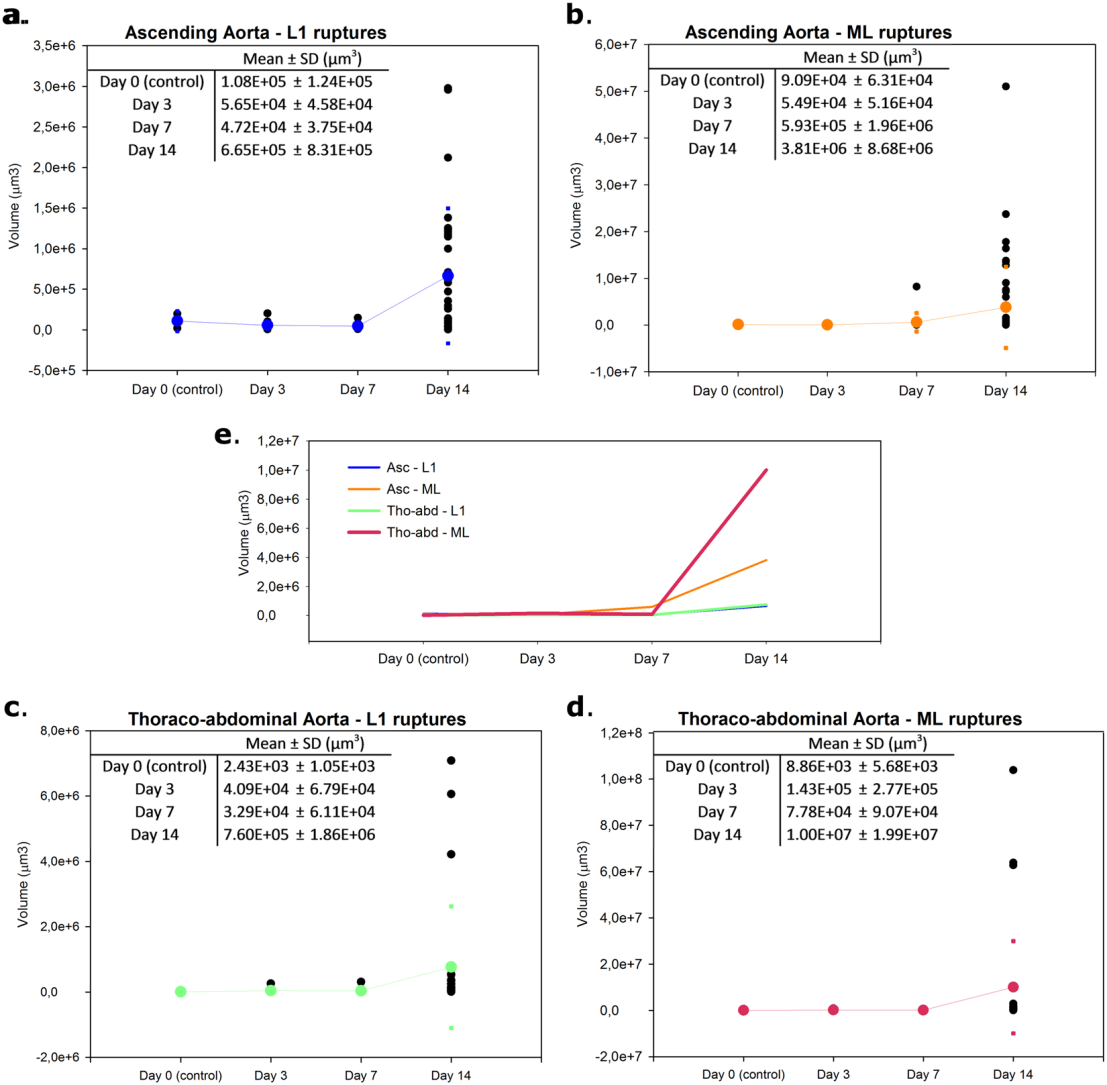
Scatter plots of volumes (in µm^3^) of the L1 and ML ruptures for ascending and thoraco-abdominal aortic samples. Volumes are depicted as black dots, mean and SD values are in blue (Ascending aorta – L1 ruptures, panel (a), orange (Ascending aorta – ML ruptures, panel (b), green (Thoraco-abdominal aorta – L1 ruptures, panel (c) and pink (Thoraco-abdominal – ML ruptures, panel (d). Panel (e) depicts the mean value of the L1 and ML ruptures at the different time points.

A steep increase in the number of L1 ruptures/animal in the ascending samples occurred comparing the control group (0.7 ruptures/mouse) to the 3-day treatment group (6.0 ruptures/mouse). The increase from the day 3 group to the day 14 group was much more gradual (6.5 ruptures/mouse for day 7 and 7.5 ruptures/mouse for day 14). In the thoraco-abdominal part, there was an increase from the control group (2 ruptures/mouse) to the day 3 group (4.7 ruptures/mouse) and an even more pronounced increase in the day 7 treatment group (8.3 ruptures/mouse). The number of L1-ruptures in the day 14 group decreased again (6.75 ruptures). The mean volume of the ruptures per treatment group is shown in Fig. 3. For both the ascending and thoraco-abdominal samples, there was a very steep increase in volume of the ML ruptures (Fig. 3, panel b,d and e) when going from day 7 to day 14 of BAPN/AngII treatment. The increase was smaller for the L1 ruptures (Fig. 3, panel a,c and e). The incidence of ML-ruptures increased gradually (see Table 1) in both the ascending (going from 2 ruptures in the control animals to 5.33, 8.50 and 12.75 ruptures in the day 3, day 7 and day 14 group respectively) and thoraco-abdominal aorta (going from 1 rupture in the control animals to 2.67, 3.67 and 6 ruptures in the day 3, day 7 and day 14 group respectively).

**Figure 4:**
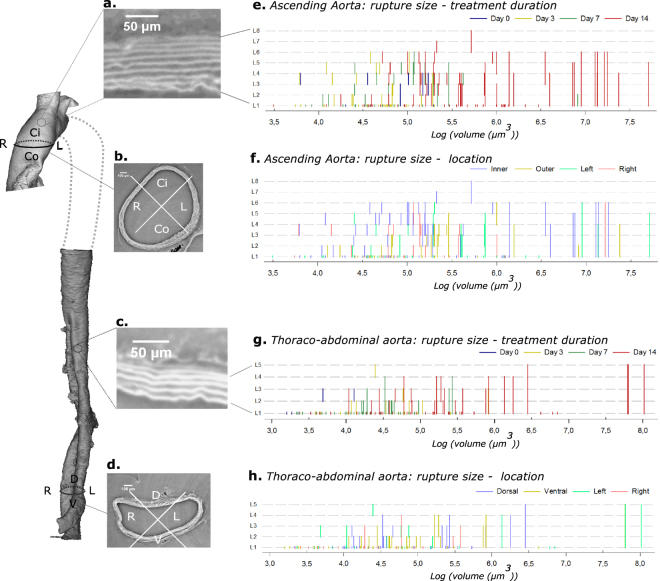
Overview of results. On the left, the resulting 3D figures of both the ascending and thoraco-abdominal aorta are shown (control mouse J3). The side panels show a portion of the pre-processed phase-contrast images (axial view). Panels (a) and (c) show a detailed portion of these images, clearly showing the individual elastic lamellae (L1-L8 for the ascending part, L1-L5 for the thoraco-abdominal part). Panels (b) and (d) show the 4 quadrants (L: left, R: right, Ci: inner curvature, Co: outer curvature, D: dorsal, V: ventral) for ascending and thoraco-abdominal portion of the aorta. On the right side, the relationship between the size of ruptures (logarithmic scale of mask volume, x-axis) and the affected medial layers (y-axis) is shown. The ruptures are grouped according to treatment in panel (f and g) (control (day 0) = dark blue, day 3 = yellow, day 7 = green, day 14 = red) and according to quadrant in panels (f and h) (Ci = blue, Co = yellow, dorsal = blue, ventral = yellow, left = green, right = red).

Figure 4 shows the quantitative analysis of the segmented ruptures in both the control animals and the BAPN/AngII animals. Panels b and d are original synchrotron images (from control animal J3) and panels a and c show a detailed portion of these images in which the individual elastic lamellae are clearly visible (up to 8 for the ascending part and up to 5 for the thoraco-abdominal part of the aorta). The elastic lamellae are represented as horizontal lines in panel e–h of Fig. 4.

In the ascending aorta, the larger L1 and ML tears occurred mostly at day 14 of BAPN/AngII treatment (Fig. 4e). The smaller tears cluster at the left side of the graph and were found throughout all stages of disease development. Most of the ML and L1 ruptures occurred in the inner segment (Fig. 4f). We did not find any ML ruptures that affected all medial layers, but we did find 6 ML ruptures that dissected 6 elastic lamellae (L1-L6, day 14) and 5 that dissected 5 layers (L1-L5, 4 at day 14, 1 at day 7).

In the thoraco-abdominal part, the largest tears were found at day 14 and smaller tears were found throughout all disease stages, similar to what was the case for the ascending aorta (Fig. 4g). The 3 largest ML and 3 largest L1 tears were located at the left and ventral side of the aorta and in vicinity of either the celiac artery or one of the intercostal arteries (Fig. 4h). These tears were found in the two samples that developed a false channel and intramural hematoma (J11 and J12 in Fig. 2). We observed 4 ML tears in which all medial layers (L1-L5) were dissected, all of them in the day 14 group.

At locations where the media was completely dissected, blood formed an intramural hematoma in between the medial and adventitial layers (J11 and J12 in Fig. 2). In J11, the false channel was located on the ventral-left side, cranially of the celiac artery. In J12, the false channel was located at the dorsal-left side at the level of the intercostal arteries. When present, the intramural hematoma was limited to the outer medial layers, did not surpass the adventitia and was more present in the cranial direction from the dissecting tears (hematoma for sample J12 is not completely shown). Presence of interlamellar blood was found in thoraco-abdominal sample J10, although no medial dissection and/or false channel was detected. There was, however, a large medial tear near the celiac artery (rupture of medial layers L1-L4) located at the ventral side, while the celiac tear in samples J11 and J12 was located at the left side of the celiac. The volumes enclosed within these tears were also substantially greater (0.06 mm^3^ for J12 and 0.10 mm^3^ for J11) than the tear in sample J10 (0.00016 mm^3^).

Even though there was no complete dissection of the media in the ascending aorta samples, there was interlamellar hematoma formation in 3 out of 4 samples from the day 14 group (Fig. 2, indicated in transparent brown). This hematoma was different from the intramural hematoma that formed in the thoraco-abdominal aorta since it was limited to the outer layers of the tunica media. Importantly, it was connected to the lumen through ruptures in the elastic lamellae (green ML ruptures in Fig. 2). In the ascending aorta of J10, 4 individual interlamellar hematomas were distinguishable (each of them connected with the lumen through multiple layer dissections), while in J11 and J12, there was only 1 large hematoma. No hematoma could be detected in sample J13. In the ascending aorta, some tears on the left side were accompanied by a symmetrical tear on the right side (Fig. 1 and Fig. 2). These paired tears were absent in control animals and early time points but occurred in 5/6 samples that were exposed to at least 7 days of BAPN/AngII treatment. In 3/6 samples one pair of these tears were found, whereas in 2/6 samples 2 pairs were found. J13 showed no presence of paired tears.

### Synchrotron scans vs. histology coupes

**Figure 5:**
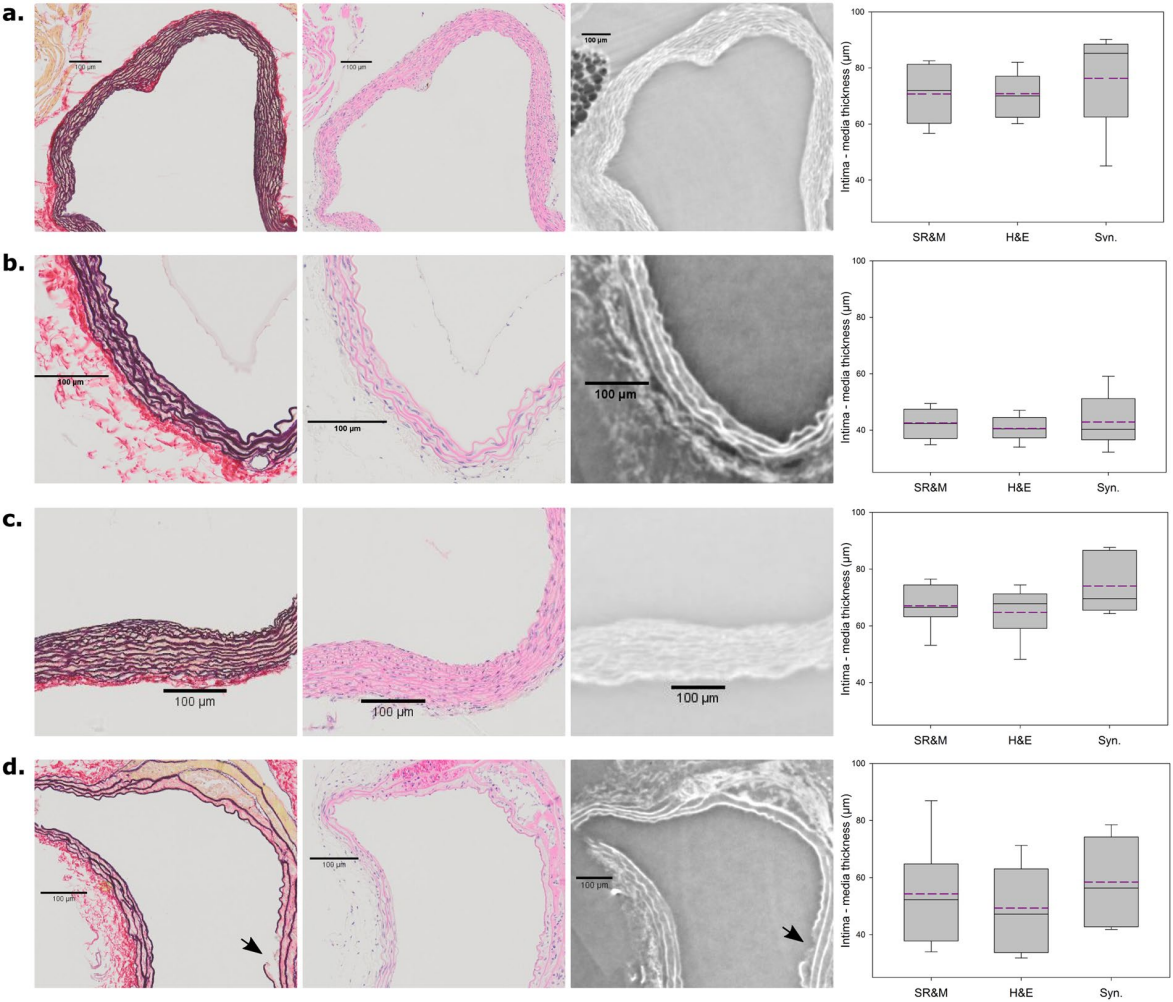
Comparison of histological staining with synchrotron scans. Left: H&E staining, middle: SR&M staining, right: synchrotron image, far right: boxplots representing the intima-media thickness (µm) measured in the SR&M, H&E and synchrotron scans (syn.). Panel (a): control (J3, ascending), panel (b): day 3 (J4, thoraco-abdominal), panel (c): day 7 (J8, ascending) and panel (d): day 14 (J12 thoraco-abdominal). The dark pink in the H&E plot of panel (d) represents an interlamellar hematoma. In the SR&M stainings, collagen is red and the elastic fibers are blue/black. Scale bar represents 100 µm. the black arrow in panel (d) shows a L1 rupture. In the boxplots minimum and maximum (horizontal lines), median, first and third quartile are shown. The purple dashed line represents the mean value of thickness.

Figure 5 shows a comparison between histological coupes and the synchrotron scans at each time point (panel a: control animals, panel b: day 3 group, panel c: day 7 group and panel d: day 14 group) for a number of representative slices. The individual lamellae could be distinguished on the histological stained slices as well as on the synchrotron scans, and other details such as laminar ruptures (Fig. 5d arrow) and interlamellar hematoma (Fig. 5d), were detectable on both the stained coupes as the synchrotron scans as well.

For the ascending aorta in the control animal, the intima-media thickness was 70.8 ± 10.5 µm in the SR&M staining, 70.8 ± 7.6 µm in the H&E staining and 76.3 ± 16 µm in the synchrotron image. The intima-media thickness of the thoraco-abdominal aorta in the day 3 animal was 42.5 ± 5.3 µm in the SR&M staining, 40.6 ± 4.1 µm in the H&E staining and 42.9 ± 8.7 µm in the synchrotron image. For the ascending part of the aorta of the day 7 animal, the intima-media thickness was 67.0 ± 7.2 µm, 64.8 ± 8.5 µm and 74.0 ± 9.4 µm for the SR&M staining, H&E staining and synchrotron image, respectively. In the thoraco-abdominal aorta of the day 14 animal, the intima-media thickness was 54.3 ± 17.5 µm in the SR&M staining, 49.3 ± 14.7 µm in the H&E staining and 58.5 ± 15.3 µm in the synchrotron image.

## Discussion

In this article, we used propagation-based phase-contrast synchrotron imaging for the visualization and quantification of the disruption of medial elastic lamella inside the aorta of a mouse model of aortic dissection disease. We obtained images of the complete murine aorta with an isotropic pixel size of 1.625 µm and analyzed the increase in both size and amount of medial ruptures in 3D. Phase-retrieval was performed according to Paganins’s method^29^. Phase propagation without phase retrieval would have resulted in a slightly better resolution^28^, but we opted for phase retrieval since it offers the best compromise between resolution (necessary to visualize medial ruptures) and image contrast (necessary to visualize the presence of hematomas)^28^. We must also point out that the samples were unpressurized at the moment of imaging and that this affects the morphology of the aorta and its structures: the lumen cross-sectional area is smaller, the media and adventitia are thinner and less uniform^20^, and the elastic lamellae have a larger tortuosity^30^. In future experiments, we will adapt our protocol so to include pressurized vessel measurements. Nonetheless, propagation-based phase-contrast synchrotron imaging offers many advantages in the field of study of aortic pathologies as more information (incidence, size, and location of (micro-) ruptures) can be extracted compared with conventional techniques. Our technique allowed us, for the first time, to quantify medial ruptures in the aorta of the BAPN/AngII mouse model (Table 1 and Fig. 4). The recorded images do not only allow for individual elastic layer tracking, they can also be used to precisely quantify intima-media thickness and to examine the presence of interlamellar hematoma formation in the ascending and thoraco-abdominal aorta, comparable with the information found in histologically stained coupes (see Fig. 5). It is particularly worth noting that (micro-) ruptures were detected not only in diseased but also (to a much lesser extent) in control animals. The presence of small discontinuities in the medial layers of healthy mouse aorta’s has been described before^11,31^ and is in line with prior histology-based data in the ascending aorta^19^. Interestingly, PCXTM-based contrast agent measurements were not able to detect any micro-ruptures in controls. This suggests that phase-contrast imaging is necessary to detect the smallest micro-ruptures^19^.

Our layer-specific data suggest that the transition from single-layer micro-ruptures to larger tears and eventually complete media dissections happens in a number of intermediate steps that are not very different between ascending and thoraco-abdominal aorta. After the onset of AngII + BAPN infusion, the L1 micro-ruptures increase in size and number and at a certain moment in time connect with each other resulting in larger ruptures (from start to day 7 of treatment). From then on, the L1 lesions only extend in size (day 7 to day 14 of treatment, see Fig. 3). The number of ML ruptures increases more gradually over time (from start to day 14 of treatment), while the size increases dramatically after day 7. Most of the L1 ruptures are part of a larger mural tear. However, this is not true for all L1 ruptures, e.g. in sample J10 (ascending) and J12 (thoraco-abdominal), Fig. 2. We conclude that the initial phase of medial tear development is similar between both aortic locations.

However, there are also marked differences between ascending and thoraco-abdominal lesions. In the ascending aorta, symmetrical tears on the left and right side of the aorta occur frequently (in all ascending samples at day 7 and day 14 except for sample J13), whereas this is not the case for the thoraco-abdominal samples. The ascending samples also have a more uniform and less severe phenotype^19^. We hypothesize that these differences could be related to (i) the absence of side branches in the ascending aorta and (ii) the higher number of lamellae in the ascending aorta.

In our prior work on ApoE^−^/^−^ mice, contrast agent infiltrations in the ascending aorta occurred after as soon as after 3 days of AngII infusion (the first investigated time point). This corresponds with the sharp increase of both ML as L1 ruptures that was observed after day 3 of treatment in the BAPN/AngII mouse model (Table 1). Paired symmetrical tears occurred in 5/6 ascending samples at day 7 and day 14. This is in accordance to what was found in previous research with the AngII-infused ApoE^−^/^−^ mice^19^, where it was hypothesized that if several laminar ruptures culminate into a dissection on one side of the ascending aorta, the aorta might elongate on this side resulting in increased tension on the other side. There is, however, a difference between BAPN and ApoE^−^/^−^ mice in terms of location of the ruptures. In ApoE^−^/^−^ mice the largest dissections in the ascending aorta occurred in the outer convex quadrant while little to no dissections occurred in the inner convex quadrant^19^. However, in this study, we found that all quadrants were affected by large tears. The interlamellar hematomas on the other hand, did form in the outer convex quadrant. While this observation is interesting, sample sizes in the current study are too small to draw meaningful conclusions on lesion variability. We conclude that in the ascending aorta there is no substantial difference in morphology between ApoE^−^/^−^ and BAPN/AngII-infused mice.

Similar to what was found in the ascending aorta, there was a sharp increase in both ML and L1 ruptures after 3 days of BAPN/AngII treatment in the thoraco-abdominal aorta. This is in line with recent findings by our group^5^, where contrast agent infiltrations showed the presence of micro-ruptures at day 3 of AngII-infused mice. We found the largest L1 and ML ruptures to occur on the ventral and left quadrant of the thoraco-abdominal aorta and close to the celiac and/or mesenteric aorta, confirming earlier findings^5,16^. A false channel was also located in the vicinity of these side branches, confirming the predisposition of this location described in AngII-infused mice^16^. The variability in phenotype observed in the thoraco-abdominal samples at day 14 of treatment corresponds to the earlier observation that there is a wide variety among the lesions found in the abdominal aorta after 28 days of AngII infusion^16^. At the same time we noticed that a BAPN/AngII treatment of 14 days was not sufficient to affect all animals to the same extent. This confirmed results from our prior meta-analysis in which we reported no difference in dissecting AAA incidence between BAPN and regular AngII infusion^32^. In this respect it is important to keep in mind that the dose and method of administration of BAPN also play an important role in the incidence and severity of the lesions^23^. We conclude that in the thoraco-abdominal aorta there is no substantial difference in morphology between AngII and BAPN/AngII-infused mice.

The main limitation of our study is its limited sample size. Because of the high competition for synchrotron beam time, we decided to focus on a detailed description of a limited number of samples, focusing mainly on the early time points. We also lack *in vivo* imaging data, which could have contributed to the determination of baseline information on (amongst others) blood pressure and *in vivo* geometry. However, the principal goal of this study was to explore propagation-based phase contrast imaging for *ex-vivo* imaging of the mouse aorta and to investigate the effect of BAPN administration on the formation of micro-ruptures. In future work these results will form the basis for a larger follow-up study in which phase-contrast propagation-based imaging will be used to quantify vascular damage in the onset of dissecting aneurysm formation in AngII-infused mice. Finally, the main purpose of the development and phenotyping of mouse models is to obtain a clear mechanistic insight into the development of human aortic pathologies, such as aortic dissection, intramural hematoma and aortic aneurysm. Data about the exact mechanism of (early) medial rupture formation in these mouse models could therefore be most valuable for our understanding of disease initiation in humans and contribute to disease prevention and treatment on the long term. Nonetheless, we must interpret these results with caution as the development of dissections and aneurysms in humans occurs over much longer time scales than the time needed to induce medial tears in the used mouse model, and the differences in anatomy and lamellar structure between murine and human aortas (which have 10 times more lamellar layers) implies that our results cannot be translated directly to a human setting. It must also be pointed out that the segmentation was performed semi-automatically and thus subject to potential interpretation errors by the observer. However, bias was kept to a minimum as all segmentations were carried out by the same person, thus precluding inter-observer variability in the analysis.

In conclusion, the advantages of phase-contrast imaging are (i) ultra-high resolution to visualize the individual elastic lamellae in the aorta; (ii) quantitative and qualitative analysis of rupture size and amount in the medial layers; (iii) 3D analysis of the complete aorta; (iv) high contrast for qualitative information extraction, reducing the need for histology coupes; (v) earlier detection of (micro-) ruptures. We used this technique to examine a known mouse model of aortic dissection provoked by BAPN/AngII treatment and showed that large medial tears are formed from smaller micro-ruptures. We did not, however, find any important morphological differences between lesions of BAPN/AngII-infused mice and lesions from regular AngII-infused mice.

## Materials and Methods

### Mice

Thirteen 11-weeks old male C57/BL6J mice (Charles River Laboratories Japan), obtained in collaboration with the Cardiovascular Research Institute at Kurume University (Kurume, Japan), were included in this experiment. N = 3 animals (J1-J3) served as controls and n = 10 (J4-J13) mice received BAPN combined with AngII in order to create a mouse model of AD, as previously reported^22^. We modified the protocol for BAPN administration to observe the early events during the AD development. Briefly, BAPN (150 mg/kg/day) was administrated simultaneously with AngII (1 µg/kg/day) using osmotic mini pumps (Alzet model 2002) for an indicated period of time. N = 3 mice were treated for 3 days (J4-J6), another n = 3 mice were treated for 7 days (J7-J9) and n = 4 mice received BAPN and AngII for a time period of 14 days (J10-J13). After the treatment, the animals were sacrificed and aortic samples were taken and fixated in paraformaldehyde (PFA). After removing PFA by washing the samples with phosphate-buffered saline (PBS), the samples were sterilized in 70% ethanol for several days. Finally, the samples were removed of 70% ethanol, as ethanol may evaporate due to the low air pressure during the shipping by air and immersed in sterile PBS for shipping to the Paul Scherrer Institute (PSI, Villigen, Switzerland).

### Imaging

All samples were imaged at the TOMCAT beamline of the Swiss Light Source in the Paul Scherrer Institute. After positioning the detector 250 mm from the sample, we could make advantage of the produced phase-contrast enhancement. This technique is also known as propagation-based phase-contrast imaging^29^ and provides images with edge enhancement, essential in distinguishing features with low density contrast. By using an X-ray photon energy of 21.8 keV, an effective pixel size of 1.625 µm was obtained. 1501 projections were acquired per sample with an exposure time of 80 milliseconds/projection, generating a total sample exposure time of roughly 2 minutes. The samples were scanned with a 180° scan and with a field of view of 3.5 $\times$ 4 mm^2^. Phase-retrieval was not performed.

### Image processing

**Figure 6:**
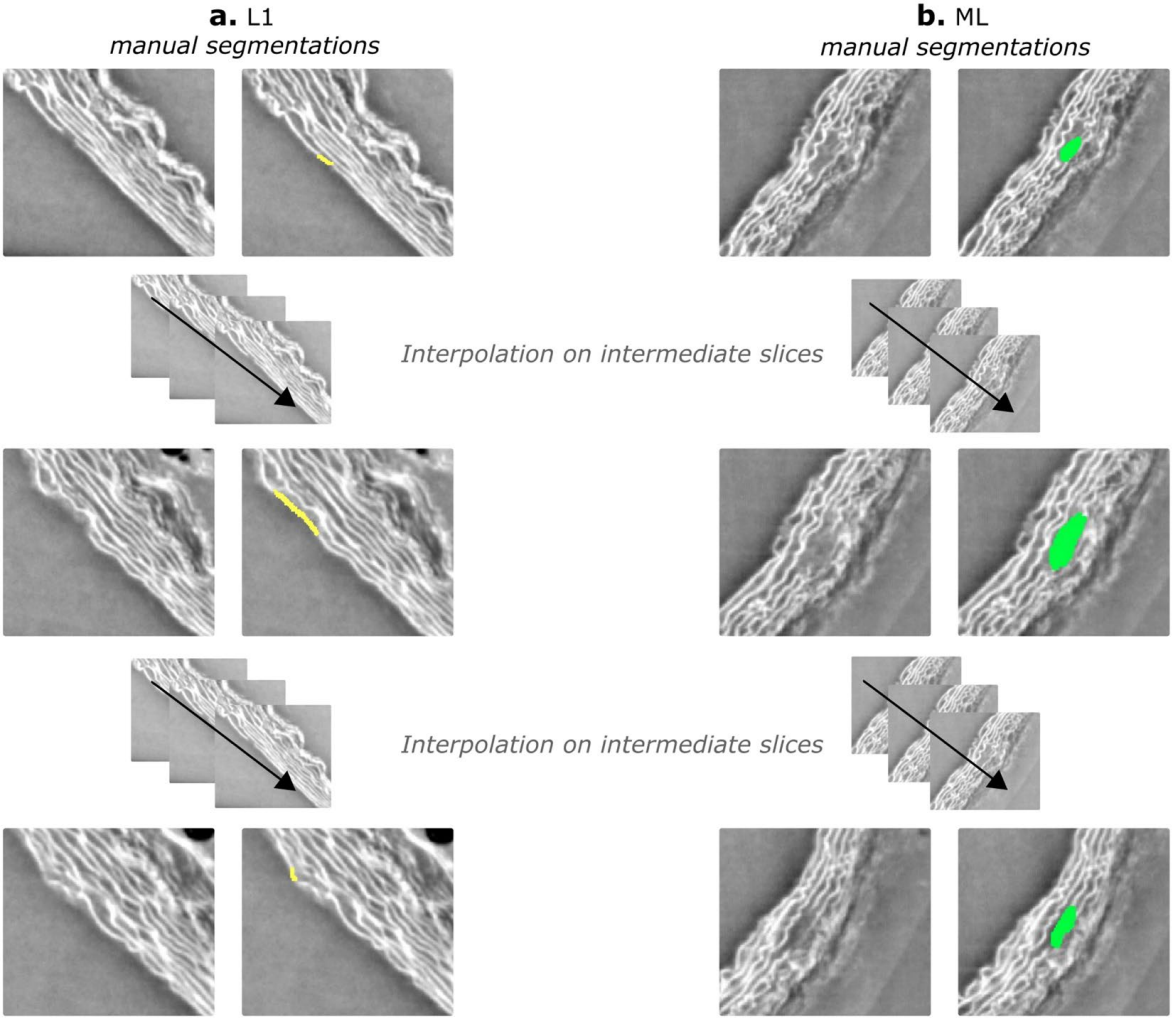
Segmentation of L1 and ML ruptures. Masks (L1 ruptures: yellow, ML ruptures: green) were created by manually segmenting the ruptures in selected axial slices) and using the Mimics interpolation tool to create the mask on the intermediate axial slices (dashed line between slices).Online resource 1 Synchrotron acquisitions of ascending aorta of sample J2 (control animal).

In the supplemental data, a movie can be found showing the synchrotron acquisitions for one sample (online resource 1). Mimics^©^ 19.0 was used for image segmentation. The ascending aortas were oriented using the coronary arteries, while in the thoraco-abdominal part the right renal and/or left suprarenal artery were used. The datasets were cropped in order to increase uniformity; Images of the ascending part were cropped to include the coronary arteries, the brachiocephalic artery and the left common carotid (not clear/present in samples J4 and J8). The image data of the thoraco-abdominal aortas were limited to include 4 pairs of intercostal arteries at the cranial side. N = 11 samples included the celiac and mesenteric aorta, but not the right renal artery (present in only n = 4 samples). Therefore, at the caudal side there were no restrictions as the celiac and mesenteric artery are known to be initiation points of medial ruptures^16^. In a next step, we made use of the ‘mask’ and ‘segmentation tools’ in Mimics^©^ 19.0 for the visualization of media, false lumen and/or intramural hematoma. Segmentation of these structures was done by using a conventional thresholding approach based on the difference in gray values. However, as the X-ray densities are very similar for the medial structures and adventitia, interruptions of the medial layers were (semi-) manually segmented: the ruptures were followed and segmented using the interpolation tool of Mimics^©^ 19.0, along the axial slices. Ruptures of the most inner elastic lamella (L1) were manually segmented with a single line, representing the absence of the elastic layer (Fig. 6a), at certain axial slices. Multiple layer ruptures (ML) (ruptures of 2 or more adjacent medial layers), were, at certain axial slices, manually segmented (Fig. 6b). The Interpolation tool of Mimics^©^ 19.0, automatically interpolated between the axial slices and the obtained segmentation was checked for irregularities. The volume of the segmented masks was calculated by Mimics^©^ 19.0. The location of the ruptures was determined in the 3D figures; 4 quadrants were defined in both the ascending and thoraco-abdominal aorta (Fig. 4b). The observed ruptures were analyzed and the following characteristics were determined: volume of missing elastic layer(s) (=volume of created masks), amount of L1/ML ruptures per sample, affected elastic layers and rupture location. In SigmaPlot™ (Systat Software, Bangalore India), a vector plot was used to visualize the volume of the L1 and ML ruptures (x-axis) with respect to the involved layers (8 for the ascending part, 5 for the thoraco-abdominal part) starting from the most inner elastic layer (L1) to the most outer ones (y-axis). A similar graph was created to visualize the volume of the L1 and ML ruptures against the affected quadrant (Fig. 4b). The mean volume of the L1 and ML ruptures in the ascending and thoraco-abdominal samples was plotted with SigmaPlot™ (Systat Software, Bangalore India).

### Histology

After imaging at the PSI, the samples were fixated by immersion in freshly prepared 4% paraformaldehyde (PFA) at 4 °C temperature for 24 hours, processed and embedded in paraffin according to standard histological procedures. 4 µm thick paraffin sections were cut just cranially of the aortic valves for the ascending aorta and at the distal end for the thoraco-abdominal samples. These sections were stained with Haematoxylin & Eosin (H&E) to assess general morphology, while Sirius Red (F3B) and Miller (SR&M) (CI35782, Direct red 80) were combined to specifically highlight elastic fibres and collagen on the same section. The according synchrotron images were used for comparison and intima-media thickness was measured in 4 quadrants (3 measurements per quadrant) in order to compensate for circumferential variability for histology plots and the synchrotron images. To measure this thickness, Fiji (Java) was used^33^ with the Action Bar plugin^34^ and BIOP plugin, developed at the EPFL (https://biop.epfl.ch/TOOL_VSI_Reader.html). SigmaPlot™ (Systat Software, Bangalore India) was used to create boxplots.

### Data availability

The datasets analysed during the current study are available from the corresponding author on reasonable request.

### Ethical approval

All applicable international, national, and/or institutional guidelines for the care and use of animals were followed. All animal experimental protocols were approved by the Animal Experiments Review Boards of Kurume University (#2017-135, #2017-091).

## Electronic supplementary material

Synchrotron images of ascending aorta
